# Sylva Americana, or Description of American Forest Trees

**Published:** 1834

**Authors:** 


### Art. VII.—Sylva Americana.

*The Sylva Americana, or a Description of the Forest Trees indigenous to the United States, practically and botanically considered; illustrated by more than* 100 *engravings.* By D. J. Browne. Boston, Hyde & Co. pp. 408, octavo.

An esteemed, but unprofessional friend, who is deeply convinced that shade trees, might be made greatly to contribute to the health and comfort of cities, has presented us with an article on the subject, and desires it to be laid before the profession. As his views coincide with our own, we of course consider them correct, and have determined not merely to place them on our pages, but to make them an occasion for directing the attention of our brethren to the botany of their native trees.

The Valley of the Mississippi embraces the most extensive and lofty forest in the United States; which in its composition, offers a greater variety of trees than any other section of the Union. Many of them are important to the physician, as being capable of furnishing valuable medicines, which might be made substitutes for the *officinals* of foreign countries; a still greater number are of value to the artisan and agriculturalist; while the whole are interesting to the botanist and the lover of nature, as she presents herself attired in the splendors of the wilderness.

Living and moving beneath the shade of these noble productions, no physician should be ignorant of their names and relations; and we would fain point out to our compeers, especially those who practise in the country, how much they lose of amusement and instruction, in their long solitary rides, (uncheered perhaps by the prospect of any good fee,) from not contemplating the woods through which they pass, with the eye of a botanist, and the heart of a poet. A slight acquaintance with the elements of the science, and the possession of a few books of practical botany, would enable them, with very little labor, to become familiar with the names, arrangement, and habitudes, of all the forest trees, beneath which they might wander, in the prosecution of their business; and thus supply them with objects for scientific remark, and much profitable reflection, on this beautiful department of organized nature, without, in any degree, diminishing their attention to the sick and the study of the profession, which should be regarded as paramount and sacred duties.

It has often struck us with some degree of astonishment, that any physician of this great valley can resist the temptation, to devote a portion of his leisure to the scientific study of its woods.

Even in winter, when leafless and shivering in the wind, they are interesting, as we then can compare the forms and mode of ramification of the different trees, both of which are the result of the manner in which the vegetative or organic energy exerts itself in the various species.

In early spring, they present still more of instruction, to him who watches the swelling bud, and observes the different influences, of the returning heat, upon different species;—some of which, like the *ulmus Americana* and the *acer rubra*, begin to expand in February, with their branches bending beneath the snow; while those of the *gymnocladus Canadensis*, hang back till the thermometer mounts up to 90° near the end of May. Curious, indeed, is this difference in the relation of the vegetative energy to caloric, in these neighboring productions of our soil. Then, again, it is interesting to notice the growth of the buds or trees of the *same* species, growing in different altitudes from the lower to the upper Mississippi, a kind of natural, more unerring in what it tells, than any artificial thermometer.

As the spring advances, the expansion of the leaves and flowers—the relative times of their appearance—and the changes of color which they undergo in opening, are new subjects of remark, and supply fresh materials for contemplation. This is the season of vegetable circulation. That of man is uninterrupted from birth to death; that of the tree revives in spring, after a long period of torpor, and then is the time for experiment and observation, on the flowing of the sap, and a comparison of that function with the circulation of the blood, an appreciation of the forces which maintain it, and a recognition of its conversion into the various secretions, which give to trees their different sensible and medical qualities.

The arrival of the solstice brings new opportunities for observing the influence of heat and moisture or drought, on the vegetative functions, in the appearance which the leaves and young fruit of the same and of different species put on, in successive seasons. It furnishes opportunities, likewise, of studying the relations which exist between this portion of the vegetable kingdom, and the great class of insects, so many of which subsist on the leaves, or deposit their ova beneath the bark of our trees. Finally, it still further discloses to us a repulsive power to the electirc fluid, in certain trees, as the beech, for instance, giving occasion, after the thunder storms which so often sweep over the woods, to observe what other species are capable of defending themselves against its lightnings.

Next comes autumn, with a wand of natural magic, transforming the forest robe of green into a hundred teints, and displaying to the eye of taste, a drapery of indescribable beauty. The approach and consummation of this change, from spring and summer verdure, to the inimitable shades of russet, yellow, orange and crimson, which the leaves put on, in the days which precede their fall, afford matter of observation and experiment, beyond what those who have not attentively surveyed the phenomenon might suppose. The influence of certain states of weather in preventing the developement of these beautiful natural dyes, and the relations which they bear to the qualities of the sap of different trees, have not, been fully revealed, and might be made the subject of a curious physiological inquiry.

Thus throughout the year, our forest presents, in every season, a new aspect and a new study, to him who can be charmed by nature in one of her most striking exhibitions of mutation; and, as these external displays arise from internal changes, the problems of vegetable physiology which they present, are highly curious, and peculiarly adapted to those who live *in sylvis*, where these beautiful phenomena of organic life, are incessantly passing before their eyes.

Nearly a hundred pages of the first part of the book before us, are devoted to the physiology of trees; but it is not our design at present to go into this subject, and if it were, this is not the book we should select for a text, as it is the work of a compiler, and designed for the general reader, rather than the philosopher. We shall, however, diversify, this *ad captandum* article, with one or two extracts, beginning with a brief account of the *root.*

"The root, which, though often differing in its bulk and shape, is similar in all its structure and use, (with the exception of the bulb, which, from containing the rudiments of a new individual, may more properly be considered a variety of the seed,) is that part of the vegetable which fixes the plant to the ground, is its organ of nourishment, and the apparatus by which, through its various ramifications, below the surface, it imbibes food from the soil.

"In its structure, it is composed of the same parts as the stem and branches, and therefore may only be considered as the stem inverted; the lower portion of the tube dipping into the earth, and forming itself into minute ramifications without leaves; and the upper portion ascending, and producing buds, branches and leaves. This has been illustrated by experiments made upon the plum, cherry and willow, in which, by inverting the stem and the root, the former has become a root, sending out ramifications, and the latter a stem producing leaves, flowers and fruit. The structure of the root and stem is therefore one and the same thing, and it is the situation in which each is placed, and the operation of the surrounding medium, that makes the difference; giving to each, a variation in its chemical and medicinal properties, though their physical structure continue the same.

"The main body of the root, which has been termed *the caudex*, upon its first penetrating the ground, possesses but very limited powers of affording nourishment; and it is not until it has sent forth its ramifications or *radiculæ*, and these ramifications have issued still finer filaments of capillary diameter, that an extensive absorption can be effected. These minute tubes, by dipping into the soil in the direction where there is the least opposition, abstract from it, by some undiscovered process, those nutritive parts, which, through the agency of water, become sap, and convey it to the caudex, from whence it rapidly ascends to the stem and branches, and thence to the uttermost extremity of the leaves, there to undergo a new modification to be hereafter explained. The root, therefore, may be considered as acting the same part towards the vegetable, as the stomach does to the animal; though the apparatus and the fluid prepared, bear but little similarity." pp. 13—14.

Our limits do not admit of our going at this time into the inquiry how the sap finds its way from the earth into the vascular system of the root, a subject which the experiments and researches of Dutrochet have contributed to elucidate. The following extract on its circulation through the plant, will not be without interest to such of our readers as have not had the means of full information on that subject.

"Much contrariety of opinion has existed among physiologists concerning the vascular system of plants, and the nature of the propulsion of the sap through their stems and branches. Indeed it is a subject upon which very erroneous ideas have prevailed.

"That the whole vegetable body is an assemblage of tubes and vessels, is evident to the most careless observer; and those who are conversant with the microscope, and books relating to it, have frequent opportunities of observing how curiously these vessels are arranged, and how different species of plants, especially trees, differ from each other in the structure and disposition of them. Such observations, however, if pursued no further, lead but a little way towards a knowledge of the wonderful physiology of vegetables.

"That plants contain various substances, as sugar, gum, acids, odoriferous fluids and others, to which their various flavors and qualities are owing, is familiar to every one; and a little reflection will satisfy, that such substances must each be lodged in proper cells or vessels, to be kept distinct from each other. They are extracted, or secreted from the common juice of the plant, and called its peculiar or secreted fluids. Various experiments and observations prove also, that air exists in the vegetable body, and must likewise be contained in appropriate vessels. Besides these, we know that plants are nourished and invigorated by the agency of water, which they readily absorb, and which is quickly conveyed through their stalks and leaves, no doubt by tubes or vessels on purpose. Finally, it is observable that all plants, as far as any experiment has been made, contain a common fluid, which at certain seasons of the year is to be obtained in great quantity, as from the vine branches by wounding them in the spring before the leaves appear, and this is properly called the sap, by which the whole body of the plant is nourished, and from which the peculiar secretions are made.

"In a young branch of a tree or shrub, or in the stem of an herbaceous plant, are found, ranged round the centre or pith, a number of longitudinal tubes or vessels, called by Mr. Knight central vessels, of a much more firm texture than the adjacent parts, and when examined minutely, these vessels often appear to be constructed with a spiral coat. This may be seen in the young twigs and leaf stalks of the elder, lilac, and many other shrubs, as well as in numerous herbaceous plants, as the peony, and more especially many of the lily tribe. If a branch or stalk of any of these plants be partly cut through or gently broken, and its divided portions slowly drawn asunder, the spiral coats of their vessels will unroll, exhibiting a curious spectacle even to the naked eye. In other cases, though the spiral structure exists, its convolutions are scarcely separable at all, or so intermediate as to be only marked by an interrupted line of perforations or slits, as shown by M. Mirbel. Indeed, the very same branches which exhibit these spiral vessels when young, show no signs of them at a more advanced period of growth, when their parts are become more woody, firm and rigid. No such spiral-coated vessels have been detected in the bark at any period of its growth.

"Besides the central vessels, Mr. Knight has described another set that traverse the alburnum, whence they are distinguished by the name of alburnous tubes. Through them the sap also ascends; for the destruction of a circle of bark does not prevent the formation of buds and leaves; 'but,' says Mr. Knight, 'the alburnous vessels appear to be also capable of an inverted action, when it becomes necessary to preserve the existence of the plant.' The cortical vessels of Mr. Knight, which can hardly be considered the same with the *vasa reducentia* of Willdenow, (although they are said to perform the same function,) exist in the bark, and serve to reconvey the circulating sap to the root. It is suspected, that there may be two sets of these vessels, one which nourishes the bark, and another that secretes particular fluids in the bark. Lymphatic vessels have also been described; but we have met with no satisfactory account of them.

"The functions of the vessels of plants have been as variously described as the organs themselves. Malpighi supposed them to be air vessels; Grew declares, that they sometimes contained moisture; and Du Hamel suspected that they contained 'highly rarified sap.' The experiments of Darwin and Knight have, to a certain degree, determined their uses. The former placed twigs of the common fig tree into a decoction of madder, and on taking them out after some hours' emersion and cutting them across, the colored fluid was found to have ascended into each branch, and the cut ends of the vessels formed a circle of red dots around the pith, and these vessels again were surrounded by other vessels containing the milky juice, so very remarkable in the fig tree. The latter (Mr. Knight) made similar experiments with cuttings of the horse chesnut and of the apple tree, with an infusion in water of very black grapes. The result corresponded with those of Darwin. He, however, pursued the investigation still further, and traced the fluid into the leaves; and during the whole course, it did not give the slightest tinge to the bark, nor to the sap between it and the wood. The pith was very slightly, if at all affected. The radicles are probably elongations of these vessels which absorb the proper fluids from the earth, and convey it into the body of the root, where it becomes sap by some process which we cannot develop; it is then conveyed to the stem and leaves, where certain other changes take place, that are to be hereafter noticed. The functions of the alburnous vessels appear to be two-fold, according to the views of Mr. Knight. At one period, they convey sap to the leaves in common with the central vessels: and during the winter, they serve as reservoirs of the juices of the plant, which, after having undergone certain changes in the leaves, are there deposited until the approach of spring, when they contribute to the formation of those new parts which are necessary for the vital action of the vegetable.

"The cortical vessels seem to carry the sap back to the roots through the bark, and, in its course, it possibly forms alburnum, or at least furnishes the materials. All this, however, is a mere probability, as we know very little with certainty connected with it.

"The ascent of the sap varies according to the season of the year, and the state and temperature of the atmosphere; being suspended during the winter, and most active in the spring, when vegetation recommences, and previously to the full expansion of the leaf; that at the vernal season, Dr. Hales has ascertained by experiments on the vine, in the heat of the day it will rise in glass tubes adjusted for the purpose, at the rate of an inch in three minutes, and attain in these tubes the height of more than twenty feet; and that, by its force upwards, it will sustain a column of mercury, of thirty-eight inches, equivalent to the pressure of a column of water of more than forty-three feet; which force, he says is, 'five times greater than that of the blood in the crural artery of a horse, seven times greater than that of a dog, and eight times greater than the blood's force of the same artery in a fallow deer.

"It is difficult to determine by what means the sap is propelled through the vessels: the agitation of the winds, the form of the vessels, the action of the heat, the pressure of certain plates, called silver grain, in the oak, are all supposed to contribute to this end; and very possibly they do this to a certain extent. We confess, however, that they do not appear to our minds adequate causes. It is a matter of some moment to ascertain how the function is performed; but our knowledge of facts is so very imperfect, that it is impossible to frame any reasonable hypothesis on the subject. In this, as in every other department of physics, men are too prone to step beyond the limits within which their actual knowledge should confine them." pp. 27—28—29—30.

In glancing over the descriptive pages of the book before us, we find that they are substantially little else than, a translation of the *History of North American Forest Trees* of Michaux, a work embellished with beautiful plates, and colored to nature—almost the whole being the result of his personal researches in various parts of the Union, nearly forty years ago. In reading several articles on the SYLVA of the western states, little known at the time they were explored by Michaux, and, apparently, not visited since by our author, we observe a number of mistakes and omissions, which it should be the business of our western botanists to correct and supply. As a specimen of this portion of the work, we shall extract a part of the article *Cornus Florida*, or Dogwood, as not only being one of our most interesting medicinal trees, but that which furnishes the *vignette* of our *Back Woods' Quarterly*.

"The wood is hard, compact, heavy and fine-grained, and is susceptible of a brilliant polish. The sap is perfectly white, and the heart is of a chocolate color. This tree is not large enough for works which require pieces of considerable volume: it is used for the handles of light tools, such as mallets, small vices, &c. It is employed by engravers for cuts used in printing. Some farmers select it for harrow teeth, for the hames of horses' collars, and also for lining the runners of sledges; but to whatever purpose it is applied, being liable to split, it should never be wrought till it is perfectly seasoned. The shoots when three or four years old, are found proper for the light hoops of small, portable casks. In the middle states, the cogs of mill wheels are made of dogwood. Such are the profitable uses of this tree; it affords also excellent fuel, but it is too small to be brought into the markets of the cities. The liber of this wood is extremely bitter, and proves an excellent remedy in intermitting fevers. The bark of this wood has a close analogy to the Peruvian bark, and has proved, in many cases, to be capable of supplying its place with success. We are told of a respectable physician of Pennsylvania, who, during twenty years, had constantly employed it, and who estimated 35 grains of it to be equivalent to 30 grains of the Peruvian bark. The only inconvenience accompanying its use was that, if taken within a year after being stripped from the tree, it sometimes occasions acute pains in the bowels: but this evil was remedied by adding to it 5 grains of Virginia snake root, *Aristolochia serpentaria*. The bark may be substituted for gall nuts, of which an excellent ink may be made by putting one-half of an ounce of it with 2 scruples of sulphate of iron, 2 scruples of gum arabic, and 16 ounces of rain water. By shaking the infusion well together it will be fit for use in a few days." pp. 142—43.

The third and last part of Mr. Browne's work consists of instructions for the culture of forest trees, which extend through nearly a hundred pages. They are minute, and appear to be judicious. In the eastern states, these directions are more important than in the western, where the destruction—not the reproduction of trees, is the *desideratum*. This, however, is only true of the more recently settled parts, for in many portions of Kentucky, Tennessee and Ohio, the waste of forest trees has been so unrelenting, that already the children of the first settlers begin to feel the want of them. It is time that every man of taste and science, began to raise his voice against a practice, which goes to deprive our country of its most grand and beautiful natural ornaments, augments the heat of its surface, diminishes the quantity of rain, promotes evaporation, and exposes both man and beast, to the fierceness of a burning sun, the moment they go abroad in summer. There is reason, moreover, to believe, that the leaves of trees exert a corrective influence on the atmosphere when vitiated, with certain noxious gases; and although this may be matter of uncertainty, it is obviously better to act on this theory, than to violate it, especially when the violation is attended with many palpable disadvantages of a different kind. But we must come to the article of our correspondent.

---

*To the Editor of the Western Journal of the Medical and Physical Sciences.*

SIR,—If in your hygienic suggestions to the public, you can recommend, professionally, the introduction of shade trees into our cities and villages, the following rules for transplanting, may not be out of place in your valuable Journal.

There are many reasons in favor of the general introduction of shade trees into our towns, but in this age of "hot-haste" in the pursuit of wealth, they do not seem sufficiently powerful to win the attention of the great mass. The embellishment which shade trees impart to towns and villages, the comforts which they confer, and the kindly influences which they exercise over the moral feelings, are among the reasons which might be urged in their favor. He who has visited New Haven, and some other of the beautiful New England villages, will at once appreciate the force of the first of these reasons: he who has wandered from the hot pavements of a city, to the cool shade of the woods, with the mercury ranging from 90 to 100°, cannot, even amid the frosts of October, be unmindful of the second; he who in midsummer, passes from the vexatious marts of traffic, to the bosom of his family grouped beneath the green foliage of the trees that overshadow his dwelling, is no stranger to the tranquillizing and purifying influences which these noble productions of nature shed upon his heart. Strong, however, as are these inducements for the cultivation of trees, they do not, here in the West, where we wage the same war of extermination upon them that we do upon the aborigines, move us to action. Now, Mr. Editor, if you could conscientiously promise us a diminution of cholera and fever, from the introduction of shade trees, it would undoubtedly promote the good cause of arboric culture. And under the belief that hygiene that will warrant such a recommendation, and that your medical brethren throughout the Valley of the Mississippi, may exert their influence in aid of the work which I am advocating, the following rules, for the successful transplanting of trees, have been collated from the ablest writers upon the subject.

1. Care should be taken in transplanting, to select those trees which have been most exposed to the elements, and conquently possess the greatest thickness and induration of bark.

2. The soil into which trees are transplanted, should be similar to but of a *better* quality, than that from which they are taken.

3. The soundness of the stem and the number and healthy condition of the roots, fibres, and rootlets, must not be overlooked.

4. Such trees should be selected as possess thrifty, and well balanced branches.

5. The practice of lopping off the branches is injudicious. When the top is too large for the roots, it should be reduced by cutting off some of the branches close to the stem.

6. The greater the amount of roots and branches retained, the better.

7. The pits in which the trees are placed, should be so large as to receive the roots in their natural position.

8. The tree should be firmly secured in its new position.

9. The best periods for transplanting in this country, are the months of October and November, March and April.

10. By an observance of the foregoing rules, trees of almost any dimensions and of every variety, may be successfully removed.

Here in the West, experience has shown that too much reliance has been placed upon the flowering locust. When in bloom it is a beautiful tree, but it affords less shade than many others, is easily broken down by the wind, and is often destroyed by insects. It is believed that not more than one-fifth of the locust trees transplanted in our city within the last ten years are now living. Our forests present a number, with superior claims to the locust. Among these may be enumerated the elm, the sugar maple, the sycamore, the buckeye, the black walnut, catalpa, and water maple. The dogwood and red bud might be added by way of variety, in gardens and public promenades. These are all indigenous. The weeping willow, the white mulberry, and the lombardy poplar, are each handsome, and may claim, although exotics, a place among our native shade trees.

A Backwoodsman.

